# Detraining’s Effects on Cardiorespiratory Fitness and Maximal and Explosive Strength in Army Soldiers: Does Age Matter?

**DOI:** 10.3390/sports12070183

**Published:** 2024-07-01

**Authors:** Alexis Arce-Álvarez, Ángelo Zaio, Camila Salazar-Ardiles, Cristian Álvarez, Pablo Merino-Muñoz, Manuel Vasquez-Muñoz, Mikel Izquierdo, Mauricio Castro, David C. Andrade

**Affiliations:** 1 Escuela de Kinesiología, Facultad de Odontología y Ciencias de la Rehabilitación, Universidad San Sebastián, Santiago 7510157, Chile; alexis.arce@uss.cl; 2 Laboratorio de Fisiología del Ejercicio y Metabolismo, Escuela de Kinesiología, Facultad de Medicina, Universidad Finis Terrae, Santiago 7501015, Chile; zaioangelo@gmail.com (Á.Z.); mcastro@uft.cl (M.C.); 3 Exercise Applied Physiology Laboratory, Centro de Investigación en Fisiología y Medicina de Altura (FIMEDALT), Departamento Biomedico, Facultad de Ciencias de la Salud, Universidad de Antofagasta, Antofagasta 1270300, Chile; nickolsalazar@gmail.com; 4 Exercise and Rehabilitation Sciences Institute, School of Physical Therapy, Faculty of Rehabilitation Sciences, Universidad Andres Bello, Santiago 7591538, Chile; cristian.alvarez@unab.cl; 5 Núcleo de Investigación en Ciencias de la Motricidad Humana, Universidad Adventista de Chile, Ñuble 3780000, Chile; pablo.merino@usach.cl; 6 Biomedical Engineering Program, COPPE, Federal University of Rio de Janeiro, Rio de Janeiro 21941-853, Brazil; 7 Dirección de Docencia de Especialidades Médicas, Dirección de Postgrado, Facultad de Medicina y Ciencias de la Salud, Universidad Mayor, Santiago 7500994, Chile; manuel.vasquez@umayor.cl; 8 Navarrabiomed, Hospital Universitario de Navarra (CHN), Universidad Pública de Navarra (UPNA), IdiSNA, 31006 Pamplona, Navarra, Spain; mikel.izquierdo@gmail.com

**Keywords:** exercise adaptations, military, tactical athletes, cardiorespiratory exercise testing, VO_2_max

## Abstract

Purpose: This study investigated the impact of four weeks of age-dependent detraining on army soldiers’ cardiorespiratory fitness and maximal and explosive strength. Methods: Fourteen volunteer tactical athletes participated, divided into two age groups (20 to 29 and 30 to 40 years). Before and after the detraining period, we assessed their anthropometric measurements (weight, height, body mass index, fat mass, and fat-free mass), cardiorespiratory fitness (maximal oxygen uptake [VO_2_max] and ventilatory thresholds [VT1 and VT2]), and kinematic properties during a single-leg counter-moving jump (CMJ) test for both the dominant and non-dominant legs. Two-way ANOVA followed by the Holm–Sidak post hoc test was used. Results: The anthropometric and cardiovascular variables did not show significant differences between the groups. However, both groups exhibited a significantly reduced maximum time and speed at the VO_2_max. Furthermore, the flight time and maximum height during the CMJ significantly decreased in the non-dominant leg for both age groups. Notably, the dominant leg’s concentric impulse (CI) significantly reduced during the CMJ, but this effect was observed only in the 30–40 age group. There were significant differences between the two age groups. Conclusions: Our findings suggest that four weeks of detraining negatively impacts aerobic fitness and muscular strength, independently of age. However, the dominant leg may be more susceptible to detraining effects in army soldiers aged 30–40. Furthermore, as a perspective, our results strongly suggest that a detraining period could affect successful missions (aerobic performance deterioration), as well as promote a muscle imbalance between the legs, which could encourage muscle injuries and endanger combat missions.

## 1. Introduction

Detraining is a well-established phenomenon, defined as the loss of training-induced performance adaptations due to reduced or ceased exercise. These losses become evident during total or partial rest periods, often caused by injury, illness, or off-season breaks [1,2]. Specifically, detraining leads to declining physiological adaptations acquired through regular exercise. Even short-term detraining can significantly impact several physiological parameters, including maximal oxygen uptake (VO_2_max), blood volume, stroke volume, and maximal cardiac output, contributing to reduced endurance performance [1]. Chen et al. (2022) [3] demonstrated that just two weeks of detraining can notably diminish cardiorespiratory fitness and muscle strength in endurance athletes. In addition, four weeks of detraining was sufficient to reduce neuromuscular performance, assessed through electromyography and jumping performance [4]; however, not all evidence demonstrates that short-term detraining is enough to promote a significant reduction in exercise performance [5,6]. Thus, while it is reasonable to hypothesize that short-term detraining could negatively affect exercise performance, the extent of this impact likely varies between populations (endurance athletes, healthy subjects, soccer players, and the army, among others).

Most detraining research has focused on endurance athletes, soccer players, and physically active people. However, limited data exists on the effects of detraining on army soldiers whose occupational performance heavily relies on physical capabilities [7,8]. These professionals require tailored training strategies to optimize job performance [9,10]. Although army soldier training incorporates general strength, grip strength, speed, skill development, mobility, and stability training [9], a traditional sports model does not apply. Instead, peak physical fitness must be maintained throughout careers to handle high-stress situations [9,10], which could be affected by age [11]. It is known that army soldiers have limited rest periods to legal holidays (rigorously controlled time); however, there is a lack of data on the effects of age-dependent detraining on this population. Moreover, stopping training in army soldiers could adversely affect successful missions and promote muscle imbalance, increasing the probability of muscle injury. Therefore, considering the controversial evidence regarding the detraining phenomenon in the non-army population [3,4,5] and the lack of evidence in soldiers across ages, our study aimed to investigate the impact of four weeks of age-dependent detraining on cardiorespiratory fitness and explosive strength in army soldiers. We sought to determine how detraining influences anthropometric measurements, cardiorespiratory fitness, and kinematic properties during single-leg countermovement jumps. We hypothesized that a 4-week detraining period could promote a significant reduction in cardiorespiratory fitness and biomechanics during single-leg jump tests in army soldiers.

## 2. Materials and Methods

### 2.1. Subjects

The subjects were recruited at the Military Physical Education Center of the Chilean Army according to the inclusion and exclusion criteria. The research design and measurements to be carried out were explained to those selected in person and subsequently invited to participate voluntarily. The military jobs consisted of administrative tasks, day and night guards in turn mode, as well as physical training. However, when military personnel are in evaluation periods (for this study, before and after detraining), they limit their work activity to administrative tasks by direct order of their instructors. Fourteen army soldiers (10 men and 4 women; 2 h/session per week [5 days]) with an average of 20 h of weekly training per day participated and were classified into two groups: 20–29 years (*n* = 7 (2 women), aged 25.88 ± 2.10 years) and 30–40 years (*n* = 7 (2 women), aged 32.83 ± 3.19 years). The inclusion criteria were (i) having > 4 years of continuous military physical training experience; (ii) being military institutionally certified as “very good” on physical tests; (iii) having no injuries/disabilities; and (iv) taking no supplements/medications. Forty-eight hours before testing, the participants avoided alcohol, smoking, caffeine, and medications. None had personal/family cardiac, respiratory, or endocrine disorders or took medications. The participants provided informed consent approved by the Universidad Mayor Ethics Committee and completed a health questionnaire. The protocols were approved by the Universidad Mayor Ethics Committee (019/2019) in accordance with the Declaration of Helsinki.

### 2.2. Design

A longitudinal study assessed the effects of four weeks of detraining on health/physical performance-related outcomes such as cardiovascular performance (the cardiopulmonary exercise test (CPET), maximum isometric strength, and explosive strength (the single-leg counter-moving jump (CMJ) test)) for dominant/non-dominant legs. Despite extensive experience (>5 years), the participants received instructions on proper, safe movement execution before testing. Assessments occurred on two separate days with 48 h between evaluations. Anthropometric measurements and the CPET were performed on day one, and the maximal isometric strength and CMJ test on day two. Due to the impossibility of monitoring the training during the legal rest period, the subjects were instructed not to follow the training plan for the 4 weeks after the first evaluation.

### 2.3. Methodology

#### 2.3.1. Anthropometrics

Anthropometrics were measured in the morning after urination while wearing underwear. The measurements followed the International Society for the Advancement of Kinanthropometry (ISAK)’s restricted profile protocol using the recommended equipment [12]. A trained anthropometrist (ISAK level 3) ensured protocol compliance. The triceps, biceps, subscapular, abdominal, supra-iliac, thigh, and calf skinfolds were measured.

#### 2.3.2. Cardiopulmonary Exercise Test (CPET)

Prior to the CPET, the participants started a warm-up that consisted of articular movement, and 6 static stretching (SS) exercises with an intensity equivalent to 5–6 on the 10-point Borg subjective perception scale (10 points) [13]. The SS exercises were intended for specific muscle groups (i.e., hamstrings, quadriceps, gastrocnemius, soleus, adductor, and psoas iliac) with a total duration of 5 min. Afterward, the participants performed a ramp CPET following a modified Åstrand protocol on a Lode Conrival cycle ergometer (Lode B.V., Groningen, The Netherlands) with customized seat/bar height adjustments. The CPET VO_2_max and ventilatory thresholds 1 (VT1) and 2 (VT2) measurements followed established protocols [14,15]. Pre-testing (after warm-up) consisted of 5 min of seated rest and then a 25 W 5 min warm-up. The test began at a 50 W resistance for men (25 W for women), increasing to 25 or 50 W every 2 min until the rpm dropped below 60 for >5 consecutive seconds. Another criterion to stop the test was to not increase heart rate between one stage and the next stage. The gas analysis used a Quark CPET system (COSMED, Rome, Italy). The VT1 was calculated in the expired VCO_2_ vs. inspired VO_2_ plot as the intersection of the two linear segments when the V_E_/VO_2_ began increasing after a plateau, and the V_E_/VCO_2_ was flat or decreasing [14,15]. The VT2 was determined in the V_E_ vs. VCO_2_ plot as the point where the V_E_/VO_2_ and V_E_/VCO_2_ began increasing [14,15]. The system was calibrated before each trial with certified gas mixtures (15% O_2_, 5% CO_2_, and balanced N_2_; Carburos Metálicos, Barcelona, Spain). The flowmeter calibration used a 3 L syringe. The V_E_/VCO_2_ began increasing after a plateau.

#### 2.3.3. Single-Leg CMJ Test

The explosive strength of each leg was assessed via the single-leg CMJ test on a force plate. Twenty-four hours prior to the measurement, the subjects were familiarized with the test, considering that it is a test that is part of the evaluations to control the military soldiers’ performance. Prior to the single-leg CMJ test, the participants were subjected to a warm-up focused on optimizing their performance during the single-leg CMJ test [13]. The participants performed 3 sets of 8 CMJs and 2 sets of 8 drop jumps (DJs) from 60 cm (DJ60), with 1 min between sets and exercises. The entire warm-up protocol lasted 5 min. The test started with the participants’ hands on their waists; the participants were instructed to “go down quickly” and “jump up as high as possible”, aiming for maximal height while raising the non-jumping leg. After a 5 min cycling warm-up, the participants practiced CMJs before 3 attempts per leg separated by 60 s rests. The dominance was defined using self-reporting and kicking ball tests, similar to what has been previously described in [16]. The highest jump was analyzed using validated biomechanical variables [17,18,19,20]. The data were processed in Matlab R2022a software (version 9.12).

#### 2.3.4. Maximal Isometric Muscle Strength

Before the maximal isometric muscle strength test, the participants were subjected to a specific warm-up [13], which consisted of submaximal isometrics exercise (1 set of 6, 4, and 2 repetitions of 50, 70, and 80% of perceived max. with one minute of resting between sets). The test consisted of participants performing 3 unilateral knee extension trials at 90° on a leg press with a force plate (Tesys 1000, Globus System, Miami, FL, USA) with 1 min inter-trial rests. The peak value was used for analysis.

#### 2.3.5. Statistical Analysis

The data of the subjects were divided according to two age groups, 20–29 and 30–40 years. The data was expressed as means ± standard deviation (SD). The normality of the data was assessed using the Shapiro–Wilk test, and the homoscedasticity of the variance was determined by Levene’s test. For the analysis between groups and pre-post detraining measurements, we used a mixed two-way ANOVA, with a factor intra-subject (time) and a factor between subject (age group), followed by the Holm–Sidak post hoc test. In addition, we used the Wilcoxon matched-pairs signed-rank test to determine the differences in pre- vs. post-conditions for each group (GraphPad Prism software Inc., version 10.0.3, La Jolla, CA, USA). The alpha value was set at *p* < 0.05. The size of the effects was calculated through η^2^ using the F-value and degree of freedom.

## 3. Results

### 3.1. Effects of Detraining on Baseline Parameters and Maximal Isometric Strength

Table 1 shows demographic and anthropometric variables before and after the 4-week detraining period for both age groups. Prior to detraining, there were no between-group differences, except for age (*p* < 0.05; Table 1), which was the criterion for dividing the tactical athletes. Four weeks of detraining did not significantly alter weight, BMI, fat mass, fat-free mass, or isometric strength compared with pre-detraining values or between groups (*p* > 0.05; Table 1).

### 3.2. Effects of Detraining on Cardiorespiratory Fitness

**Figure 1:**
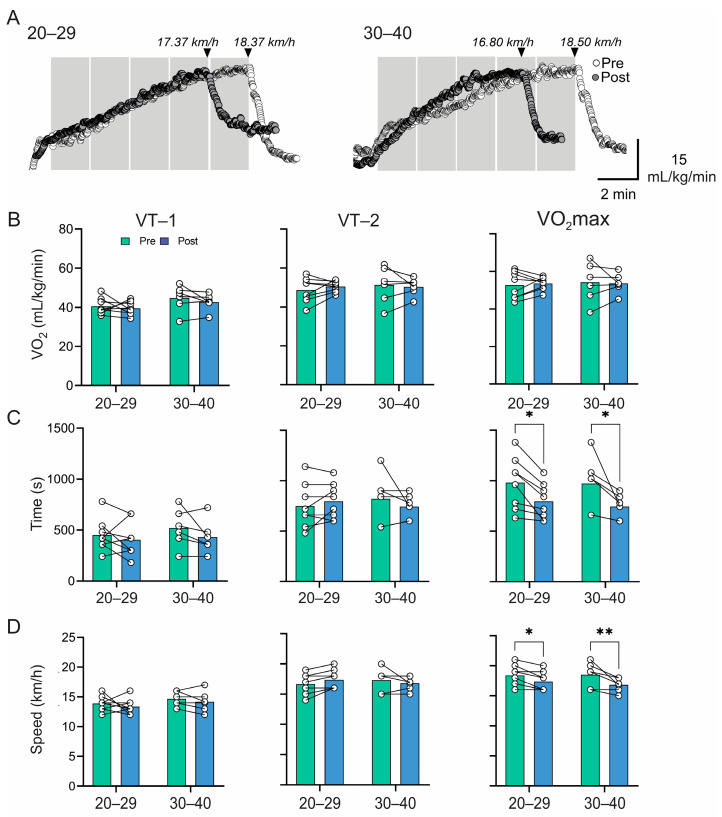
Cardiovascular response during a maximal incremental test. (**A**) Representative VO_2_ recordings during the maximal effort test of army soldiers from the 20–29 age group and one from the 30–40 age group. (**B**–**D**) Summary data of oxygen consumption, total time, and velocity at VT1, VT2, and VO_2_max. Note that four weeks of detraining worsened time performance in the maximal test without changes in army soldiers’ cardiorespiratory capacities and ventilatory thresholds. Two-way ANOVA with repeated measures followed by the Holm–Sidak post hoc test and the Wilcoxon matched-pairs signed-rank test determined differences in pre- vs. post-conditions for each group in (**B**–**D**). Values are means ± SD; 20–29 *n* = 7; 30–40 *n* = 7. * *p* < 0.05; ** *p* < 0.01.

Our data revealed four weeks of detraining did not significantly impair cardiorespiratory responses at VT1 and VT2 during maximal exercise testing (Table 2). Although the VO_2_max was similar between groups (Figure 1A,B), the maximum duration (20–29 years: 795.00 ± 177.84 vs. 978.75 ± 256.04 s, (F [DFn, DFd] = 20.97 [1, 12], η^2^ = 0.64, *p* < 0.05; 30–40 years: 744.00 ± 123.54 vs. 970.00 ± 274.74 s, (F [DFn, DFd] = 20.97 [1, 12], η^2^ = 0.64, *p* < 0.05 (post- vs. pre-conditions) (Figure 1A,C)) and speed (20–29 years: 17.37 ± 1.59 vs. 18.37 ± 1.68 km/h, F [DFn, DFd] = 20.75 [1, 12], η^2^ = 0.63, *p* < 0.05; 30–40 years: 16.80 ± 1.16 vs. 18.50 ± 2.07 km/h, F [DFn, DFd] = 20.75 [1, 12], η^2^ = 0.63, *p* < 0.05 (post- vs. pre-conditions) (Figure 1A,D)) during the CPET significantly declined in both groups, without between-group differences (Figure 1).

### 3.3. Effects of Detraining on CMJ Performance, Kinetic and Kinematic

**Figure 2:**
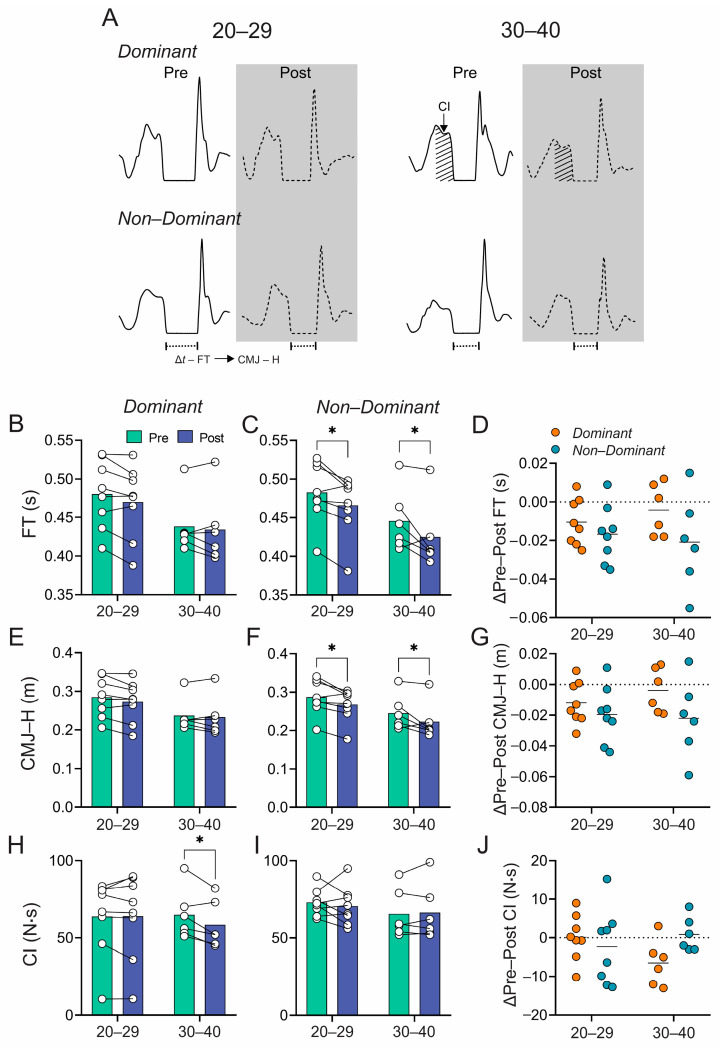
Jump performance and biomechanical properties during single-leg counter-moving jump (CMJ) test for dominant and non-dominant legs. (**A**) Representative recordings of countermovement jump for the dominant and non-dominant legs of a subject in the 20-29 age group and a subject in the 30–40 age group. (**B**,**C**) Summary data of flight time (FT) pre- and post-detraining of dominant and non-dominant legs, respectively. (**D**) ΔFT of the dominant and non-dominant legs of the 20–29 and 30–40 age groups. (**E**,**F**) Summary data of countermovement jump height (CMJ-H) pre- and post-detraining of dominant and non-dominant legs, respectively. (**G**) ΔCMJ-H of the dominant and non-dominant legs of the 20–29 and 30–40 age groups. (**H**,**I**) Summary data of concentric impulse (CI) pre- and post-detraining of dominant and non-dominant legs, respectively. (**J**) ΔCI of dominant and non-dominant legs of the 20–29 and 30–40 age groups. Note that the effects of detraining are markedly significant in the non-dominant leg for both groups. Two-way ANOVA with repeated measures followed by the Holm–Sidak post hoc test and the Wilcoxon matched-pairs signed-rank test to determine differences in pre- vs. post-conditions for each group in (**B**–**G**). Values are means ± SD; 20–29 *n* = 7; 30–34 *n* = 7. * *p* < 0.05.

Table 3 and Figure 2 show the detraining-induced changes during the single-leg CMJ test. Four weeks of detraining significantly decreased flight time (20–29 years: 0.46 ± 0.04 vs. 0.48 ± 0.04 s, F [DFn, DFd] = 13.08 [1, 12], η^2^ = 0.52, *p* < 0.05; 30–40 years: 0.42 ± 0.04 vs. 0.45 ± 0.04 m, F [DFn, DFd] = 13.08 [1, 12], η^2^ = 0.52, *p* < 0.05 (post vs. pre-conditions) (Figure 2A,C)) and jump height (20–29 years: 0.26 ± 0.04 vs. 0.29 ± 0.04 m, F [DFn, DFd] = 13.05 [1, 12], η^2^ = 0.52, *p* < 0.05; 30–40 years: 0.22 ± 0.05 vs. 0.25 ± 0.04 m, F [DFn, DFd] = 13.05 [1, 12], η^2^ = 0.52, *p* < 0.05 (post- vs. pre-conditions) (Figure 2A,F)) in the non-dominant leg in both groups, without between-group differences (Figure 2D,G). The dominant leg’s flight time and jump height did not differ between groups (Figure 2B,E). However, the concentric impulse (CI) of the dominant leg significantly decreased after detraining only in the older group (30–40 years: 58.33 ± 15.39 vs. 64.83 ± 16.41 N·s, F [DFn, DFd] = 3.99 [1, 12]; η^2^ = 0.24; *p* < 0.05 (post- vs. pre-conditions, respectively) (Figure 2H)), with no between-group differences (Figure 2H–J). No other kinetic or kinematic variables differed between groups or legs (Table 3).

## 4. Discussion

The main aim of the present study was to investigate the impact of four weeks of age-dependent detraining on army soldiers’ cardiorespiratory fitness and explosive strength. The key findings were as follows: (i) While we did not find a significant deterioration in the VT1, VT2, and VO_2_max, the maximum speed and distance were significantly reduced after four weeks of detraining, independent of age. (ii) The non-dominant leg was more affected by four weeks of detraining, showing a decreased flight time and jump height during the single-leg CMJ test in both groups. (iii) The CI of the dominant leg declined after four weeks of detraining only in the 30–40-year-old group, suggesting the dominant leg may be more susceptible to detraining effects in older army soldiers. Thus, our results indicate that four weeks without training could impair maximum aerobic performance without changes in the VO_2_max in army soldiers. Additionally, we observed the non-dominant leg was more susceptible to reduced jumping performance, reflected in the decreased flight time and jump height during the single-leg CMJ test. Interestingly, these adaptative responses to short-term detraining were age-independent. However, the CI of the dominant leg only deteriorated in older army soldiers, suggesting this kinematic variable could be age-susceptible.

### 4.1. Cardiovascular Adaptations to Detraining in Army Soldiers

Aerobic capacity is critical for developing army soldiers, as cardiorespiratory performance plays a pivotal role in daily military activities. Occupational tasks relying exclusively on cardiorespiratory capacity include patrolling long distances, pursuits, and working at high temperatures. While detraining’s effects on cardiovascular capacity have been widely studied in traditional sports [5,7,21], the evidence in army soldiers is limited. We found maximal the O2 uptake to be unchanged after four weeks of detraining; however, the CPET performance markedly decreased. Controversially, Coyle et al. (1986) [21] showed three weeks of detraining decreased cardiovascular capacity in runners, mainly from reduced blood and stroke volumes [21]. Additionally, other studies have revealed that short-term detraining (1–4 weeks) induces cardiac remodeling characterized by left ventricular atrophy, potentially decreasing contractility, stroke volume, and VO_2_max [22,23]. Our results contrast with previous observations, likely explained by more research on competitive athletes with different training regimens, a stronger emphasis on cardiovascular work, variations in physical activity during follow-up, and detraining durations [21].

Importantly, army soldiers lack defined rest seasons besides mandated legal rest periods, which differ entirely from traditional sports athletes. Interestingly, our data show age was not critical for detraining-induced aerobic performance deterioration in army soldiers, as both groups displayed similar CPET performances. However, this may be because the most significant declines in trained athletes occur between 40 and 50 years [11]. Accordingly, expanding the age range in future studies could help determine the critical age at which detraining becomes relevant in army soldiers.

### 4.2. Detraining Effects on Jump Performance

Muscular strength and power strongly relate to the daily tasks of army soldiers [22,23,24]. Activities like hand-to-hand combat, lifting or moving equipment, clearing obstacles, or carrying accident victims require explosive strength. Jump performance and associated kinetics and kinematics could indicate explosive strength and reflect detraining’s detrimental effects. However, the evidence on detraining-induced explosive strength impairment in army soldiers is minimal despite extensive studies in competitive athletes across disciplines. Pereira et al. (2016) [25] found that four weeks of detraining did not affect countermovement jumps (CMJs) in runners [25], and seven weeks of detraining did not significantly alter jump height in elite handball players [26]. Our results differ from previous findings in these athletes. Indeed, we found four weeks of detraining significantly reduced jump performance in army soldiers, mainly in the non-dominant leg. One explanation may be tactical athlete training focuses on permanent readiness without competition or rest between tasks, so detraining periods correspond to legal rest. Thus, our results may differ because army soldiers underwent complete training cessation rather than a planned detraining period. However, the literature defines detraining as the partial or complete loss of anatomical, physiological, and performance adaptations from reducing or ceasing training [1,2]. In fact, planned detraining may more closely resemble a tapering process [27,28]. Therefore, our results show four weeks without training can impair jump performance and kinematics in army soldiers.

### 4.3. Limitations

This study is not free of limitations. We did not control lifestyle factors during the army soldiers’ legal rest period. Nutritional regimens, additional rest periods, and training protocols were not monitored; however, the tactical athletes were instructed not to follow any training plan during the 4 weeks after the first evaluation. Furthermore, exhaustive anthropometric measurements were not conducted to determine muscle mass changes related to CMJ performance during rest. In addition, unfortunately, our testing was not randomized, which could be a bias during the testing sessions. Furthermore, according to our data, we observed a moderate within-session reliability (intraclass correlation coefficient (ICC) of ~0.6). Regarding the number of subjects for the aerobic and jump height outcomes, the adequate sample size, with a 1-ß of 0.8, was 20 and 40, respectively; however, considering the current number of subjects (*n* = 14), the sample power was 0.63 and 0.41 with an alpha of 0.05, respectively. Thus, our number of subjects was insufficient to conclude robust effects of detraining on military personnel. However, it is essential to mention that the detraining was defined according to the tactical staff available during this period and occurred during holidays, limiting the number of subjects for the present study. Therefore, future studies should consider weekly surveys of army soldiers’ physical activity, diet, and rest during detraining, as well as increase the number of participants. Monitoring anthropometry during this time to determine muscle mass’s influence on CMJ performance would also be beneficial.

## 5. Conclusions

Our findings demonstrate four weeks without training could negatively impact aerobics and muscular performance in army soldiers, evidenced by the significantly reduced maximum speed and time during the CPET and a markedly decreased jump height and flight time during CMJ testing. These declines were age-independent. Additionally, we found that the concentric impulse of the dominant leg deteriorated more in the 30–40-year-old group, suggesting the dominant leg may be more susceptible to detraining effects in older army soldiers than younger ones. Our results strongly suggest that long-term training interruptions could impact aerobics performance, which could affect successful missions. They also suggest that this promotes a muscle imbalance between the legs, which could promote muscle injuries and endanger combat missions.

## Figures and Tables

**Table 1:** Comparison of the demographic and anthropometric variables of the groups of 20–29 and 30–40 years.

	20–29 Years (*n* = 7)		30–40 Years (*n* = 7)	
	Pre	Post	Pre	Post
Age (years)	25.8 ± 2.10	-	32.8 ± 3.19 *	-
Weight (kg)	64.8 ± 7.14	65.5 ± 7.16	60.5 ± 8.34	60.7 ± 8.48
Height (m)	1.69 ± 0.05	-	1.64 ± 0.08	-
BMI (kg/m^2^)	22.8 ± 1.42	23.0 ± 1.38	22.4 ± 1.41	22.4 ± 1.44
Fat mass (%)	22.3 ± 4.77	23.1 ± 3.96	23.4 ± 4.02	23.2 ± 3.60
Fat-free mass (kg)	50.53 ± 7.37	50.4 ± 7.15	46.4 ± 7.8	46.7 ± 8.05
Isometric strength (N)	1122 ± 251	1124 ± 221	1097 ± 252	1105 ± 295

Values are means ± SD. BMI: body mass index. Age and height were analyzed using the non-parametric Mann–Whitney and the parametric unpaired *t*-test, respectively. Weight, BMI, fat mass, fat-free mass, and isometric strength were analyzed with repeated measures ANOVA (2 × 2) followed by the Holm–Sidak post hoc test and the Wilcoxon matched-pairs signed-rank test to determine differences in pre- vs. post-conditions for each group. * *p* < 0.05.

**Table 2:** Age-related differences in cardiopulmonary variables during the maximum CPET before and after 4 weeks of detraining in army soldiers.

	VT1				VT2			
	20–29		30–40		20–29		30–40	
	Pre	Post	Pre	Post	Pre	Post	Pre	Post
VO_2_ (mL/kg/min)	2.66 ± 0.42	2.59 ± 0.48	2.72 ± 0.58	2.67 ± 0.59	3.19 ± 0.55	3.30 ± 0.46	3.12 ± 0.72	3.15 ± 0.66
V_E_ (L/min)	76. 51 ± 15.39	70.68 ± 14.80	75.32 ± 16.32	72.34 ± 20.28	100.61 ± 17.28	104.44 ± 15.18	97.80 ± 21.48	96.74 ± 25.63
IV	73.50 ± 14.70	67.81 ± 14.40	72.67 ± 16.07	69.40± 19.72	96.38 ± 16.49	100.19 ± 14.68	93.67 ± 20.91	92.60 ± 24.70
VCO_2_ (mL/kg/min)	2.20 ± 0.46	2.10 ± 0.46	2.28 ± 0.49	2.21 ± 0.57	2.79 ± 0.50	3.02 ± 0.47	2.82 ± 0.59	2.87 ± 0.66
EqO_2_	28.63 ± 3.59	27.25 ± 2.83	27.92 ± 4.03	27.00 ± 4.32	31.75 ± 3.58	31.75 ± 1.49	31.67 ± 3.22	30.80 ± 4.10
EqCO_2_	34.81 ± 3.03	33.88 ± 3.50	33.50 ± 3.39	33.00 ± 5.30	36.19 ± 4.25	34.69 ± 3.54	34.92 ± 2.89	34.00 ± 4.05
F_E_O_2_ (%)	19.15 ± 0.59	18.95 ± 0.39	18.95 ± 0.52	18.78 ± 0.76	19.52 ± 0.50	19.53 ± 0.21	19.46 ± 0.39	19.29 ± 0.57
F_E_CO_2_ (%)	3.81 ± 0.38	3.93 ± 0.38	3.99 ± 0.39	4.20 ± 0.69	3.69 ± 0.43	3.83 ± 0.38	3.81 ± 0.34	4.09 ± 0.56
V_E_/VO_2_	28.70 ± 3.60	27.33 ± 2.74	27.92 ± 4.03	27.11 ± 4.28	31.71 ± 3.57	31.69 ± 1.53	31.65 ± 3.21	30.70 ± 3.87
V_E_/VCO_2_	34.88 ± 3.11	33.80 ± 3.48	33.32 ± 3.31	33.11 ± 5.21	36.21 ± 4.12	34.79 ± 3.49	34.85 ± 2.90	33.96 ± 4.26
PetO_2_ (mmHg)	100.94 ± 4.18	100.00 ± 2.88	99.50 ± 3.90	99.20 ± 5.89	104.94 ± 3.65	105.31 ± 1.89	103.75 ± 3.88	103.70 ± 4.72
PetCO_2_ (mmHg)	34.69 ± 2.62	35.63 ± 2.99	36.17 ± 2.93	37.50 ± 5.79	32.75 ± 3.16	34.00 ± 3.23	34.67 ± 3.19	36.20 ± 4.71
RQ	0.82 ± 0.09	0.81 ± 0.07	0.84 ± 0.06	0.82 ± 0.05	0.88 ± 0.10	0.92 ± 0.07	0.91 ± 0.05	0.91 ± 0.03

Values are shown as means ± standard deviation. VO_2_: absolute oxygen consumption; V_E_: minute ventilation; IV: inspiratory volume; VCO_2_: carbon dioxide production; EqO_2_: ventilatory equivalent for VO_2_; EqCO_2_: ventilatory equivalent for VCO_2_; F_E_O_2_: expired fraction of O_2_; F_E_CO_2_: expired fraction of CO_2_; V_E_/O_2_: ventilatory equivalent to O_2_; V_E_/CO_2_: ventilatory equivalent to CO_2_; HR: heart rate; PetO_2_: end-tidal partial pressure of O_2_; PetCO_2_: end-tidal partial pressure of CO_2_; and RQ: respiratory quotient. The data were analyzed with repeated measures ANOVA (2 × 2) followed by the Holm–Sidak post hoc test and the Wilcoxon matched-pairs signed-rank test to determine differences in pre- vs. post-conditions for each group.

**Table 3:** Age and leg dominance-related differences in kinetic and kinematic variables from unilateral countermovement jump (CMJ) test before and after 4 weeks of detraining in army soldiers.

	20–29 Years (*n* = 7)				30–40 Years (*n* = 7)			
	Dominant		Non-Dominant		Dominant		Non-Dominant	
	Pre	Post	Pre	Post	Pre	Post	Pre	Post
CT (s)	0.72 ± 0.05	0.68 ± 0.10	0.74 ± 0.10	0.74 ± 0.06	0.69 ± 0.13	0.70 ± 0.11	0.71 ± 0.06	0.72 ± 0.06
CCT (s)	0.28 ± 0.04	0.27 ± 0.05	0.28 ± 0.04	0.27 ± 0.03	0.27 ± 0.03	0.26 ± 0.05	0.26 ± 0.04	0.26 ± 0.03
UT (s)	0.16 ± 0.07	0.14 ± 0.05	0.18 ± 0.07	0.18 ± 0.06	0.26 ± 0.02	0.26 ± 0.05	0.26 ± 0.04	0.26 ± 0.03
RSI-M	0.40 ± 0.07	0.41 ± 0.06	0.39 ± 0.08	0.37 ± 0.08	0.35 ± 0.09	0.34 ± 0.10	0.35 ± 0.09	0.31 ± 0.07
PF (N)	816 ± 106	783 ± 148	807 ± 67	779 ± 76	729 ± 142	690 ± 181	719 ± 144	690 ± 105
PF (N/kg)	23.13 ± 2.48	22.6 ± 3.70	24.75 ± 2.66	23.13 ± 2.70	21.67 ± 2.07	21.3 ± 2.07	22.6 ± 2.88	23.1 ± 2.48
PP (W)	1337 ± 356	1399 ± 473	1317 ± 163	1256 ± 232	1106 ± 384	966 ± 293	1150 ± 341	1218 ± 393
PP (W/kg)	37.75 ± 8.38	39.63 ± 11.14	40.50 ± 8.09	37.13 ± 6.78	32.67 ± 8.27	30.17 ± 3.76	36.17 ± 9.37	40.17 ± 8.91
P-RFD (N/s)	6189 ± 2471	5908 ± 2728	6229 ± 1538	6257 ± 1821	4062 ± 948	4431 ± 2157	4747 ± 1539	4223 ± 1100
T-P-RFD (s)	0.32 ± 0.05	0.27 ± 0.07	0.33 ± 0.05	0.32 ± 0.06	0.32 ± 0.06	0.32 ± 0.06	0.33 ± 0.04	0.33 ± 0.06
RFD-Y (N/s)	2164 ± 925	2284 ± 898	2454 ± 762	2324 ± 453	1382 ± 518	1659 ± 921	1721 ± 527	1459 ± 346
RFD-Y (N/s/kg)	61.75 ± 25.73	65.63 ± 24.42	72.88 ± 14.19	67.88 ± 8.29	40.50 ± 12.53	50.50 ± 19.60	54.00 ± 14.14	49.17 ± 13.67
T-RFD-Y (s)	0.13 ± 0.04	0.11 ± 0.03	0.12 ± 0.02	0.11 ± 0.02	0.15 ± 0.04	0.14 ± 0.05	0.13 ± 0.03	0.14 ± 0.04
RFD-B (N/s)	2966 ± 817	2790 ± 984	2906 ± 721	2648 ± 839	2489 ± 753	2465 ± 893	2512 ± 897	2369 ± 583
RFD-B (N/s/kg)	84.88 ± 25.63	81.22 ± 27.57	90.75 ± 30.70	80.38 ± 30.91	73.00 ± 14.48	76.17 ± 17.68	78.83 ± 27.01	79.17 ± 19.81
T-RFD-B (s)	0.16 ± 0.03	0.16 ± 0.05	0.17 ± 0.04	0.17 ± 0.03	0.15 ± 0.03	0.15 ± 0.02	0.16 ± 0.02	0.16 ± 0.01
RFD-E (N/s)	2560 ± 680	2521 ± 814	2623 ± 294	2472 ± 459	1938 ± 608	2080 ± 903	2133 ± 630	1919 ± 344
RFD-E (N/s/kg)	73.38 ± 21.15	73.00 ± 22.73	80.75 ± 15.17	74.25 ± 18.60	56.83 ± 12.22	63.83 ± 18.66	66.833 ± 18.41	64.17 ± 12.70
T-RFD-E (s)	0.29 ± 0.06	0.26 ± 0.07	0.29 ± 0.04	0.29 ± 0.04	0.30 ± 0.06	0.29 ± 0.06	0.29 ± 0.04	0.30 ± 0.04
EI (N·s)	33.59 ± 27.34	25.735 ± 11.08	25.74 ± 11.07	24.43 ± 4.80	13.83 ± 6.62	15.83 ± 7.25	18.17 ± 9.77	17.83 ± 4.12
S (N/m/kg)	100.63 ± 43.78	112.63 ± 45.28	90.88 ± 34.16	84.50 ± 23.30	152.33 ± 150.43	106.33 ± 39.24	97.50 ± 12.44	97.33 ± 22.97
PL (N)	1568 ± 226	1489 ± 412	1345 ± 410	1605 ± 329	1498 ± 288	1342 ± 233	1455 ± 544	1558 ± 408

Values are means ± standard deviation. CT: contraction time; CCT: concentric time; UT: unloading time; RSI: explosive strength index; RSI-M: modified RSI; FT: flight time; R-FT:CT: FT–CT ratio; PF: peak force; PP: peak power; RFD: rate of force development; P-RFD: peak RFD; T-P-RFD: time to peak RFD; RFD-Y: RFD yielding; T-RFD-Y: time to RFD yielding; RFD-B: RFD braking; T-RFD-B: time to RFD-B; RFD-E: RFD eccentric; T-RFD-E: time to RFD eccentric; EI: eccentric impulse; S: stiffness; PL: peak landing. Wilcoxon matched-pairs signed-rank test was performed on 20–29 and 30–40 groups separately.

## Data Availability

The data are contained within this article.
